# ‘There isn’t a checklist in the world that’s got that on it’: Special needs teachers’ opinions on the assessment and teaching priorities of pupils on the autism spectrum

**DOI:** 10.1177/1744629520972901

**Published:** 2020-12-16

**Authors:** Melanie Howell, Jill Bradshaw, Peter E Langdon

**Affiliations:** 2240 University of Kent, UK; 2240 University of Kent, UK; 2707 University of Warwick, UK

**Keywords:** assessment, autism, special education, special education teachers

## Abstract

Two focus groups were conducted with special needs teachers to: (a) identify barriers to learning for autistic pupils, (b) consider broad assessment domains and specific skills or behaviours which teachers consider important for these pupils, and (c) give their opinions on teacher assessments. Data analysis resulted in six main themes: (a) barriers to learning, (b) teacher priorities for autistic pupils, (c) ways of overcoming barriers, (d) the concept of ‘true mastery’, (e) assessing the bigger picture, and (f) practicalities of assessment. Results showed that teachers have priorities for the pupils they know well and concerns about the assessments they regularly use. To ensure face and content validity of teacher assessments, and for assessments to be useful to and valued by the teachers who use them, it is recommended that teachers have opportunities to input during various aspects of the assessment development process.

## Introduction

### Background

When it comes to pupils with special educational needs (SEN), teachers are often best placed to provide great insight into both the teaching and learning needs of their pupils. Although much of the research focusing on autism education is about inclusive education in mainstream schools, research specifically involving SEN teachers is becoming more common. In recent studies, for example, SEN teachers have been asked their opinions on different teaching interventions for pupils on the autism spectrum (e.g. Mills and Chapparo, 2018; Spencer, 2017) and thoughts on behaviour difficulties (e.g. Adams et al., 2019; Welsh et al., 2019). Although research has explored parent priorities for teaching and outcomes for autistic children (McConachie et al., 2018), little research has been conducted on outcomes that SEN teachers consider to be important or on SEN teachers’ views on assessment within in the United Kingdom. Azad and Mandell (2016) conducted interviews with parents and teachers of children on the autism spectrum in the USA and compared their concerns and priorities for intervention. They found that teachers’ primary and secondary concerns were ‘problem behaviour’ and ‘deficits in social interaction’ and these were also the main concerns of parents (p. 437). For teachers, these top two concerns were followed by restricted and repetitive behaviours (RRBs), communication and self-help. Teachers were least concerned about academic development. Helps et al. (1999) conducted a British study which considered mainstream and SEN teachers’ knowledge and understanding of autism. Teachers were asked about the difficulties they face when working with pupils on the autism spectrum, with the most cited difficulty being lack of knowledge, followed by repetitive or obsessional behaviours, poor communication and aggressive and self-injurious behaviours. Recent studies considering SEN teachers’ opinions on the important skills and progress of their pupils on the autism spectrum do not appear to have been conducted, particularly in the United Kingdom. Again, there appears to be little, if any, research considering SEN teachers’ views of educational assessment.

### Assessment

Special school assessment necessarily differs from mainstream assessment due to the needs of the pupils. Recent changes to assessment in England have resulted in new statutory English and maths assessment for pupils working below National Curriculum standard but engaged in subject-specific learning (Standards and Testing Agency, 2020a, 2020b). For pupils working below National Curriculum standard in secondary schools or for primary subjects outside of English and maths, there is no statutory assessment specified (Smith et al., 2020). Special school assessments prescribed by the government or developed by private companies are often created for pupils with a range of SEN rather than the specific needs of pupils on the autism spectrum. Considering the assessments that can be used within SEN contexts, few are developed in conjunction with or with input from the teachers who use the assessments day to day (Howell et al., 2020). Those that are developed with input (often minimal) from teachers are not always tested for comprehensibility or evaluated in their final form for validity and reliability. Rarely are teachers asked about the *kinds* of skills and behaviours they feel are important to assess. Similarly, assessments which are widely used in special schools in England are not often subject to rigorous evaluation of their measurement properties (Howell et al., 2020).

Within this paper, the outcomes from two initial focus groups with SEN teachers will be described and discussed. The research aims of the focus groups were: (a) to identify important areas of progress and barriers to learning for pupils on the autism spectrum, (b) to consider broad areas and specific skills or behaviours which SEN teachers think are important to assess for autistic pupils, and (c) to consider the features of assessment tools which are useful to SEN teachers in the classroom.

## Methods

The face-to-face and discursive nature of focus groups meant that participants could generate and develop ideas together and explore issues by responding to others’ contributions in real time (Morgan, 1998). The open-ended nature of discussion allowed for more detail in participant responses compared to a questionnaire or survey and the interactive element allowed participants to consider a variety of opinions, to explore and convey their priorities and address subjects they felt were most important (Detmar et al., 2006).

### Participants and procedures

The two focus groups were made up of a convenience sample of 21 teachers in total (18 female and 3 male) from two special schools in the south of England. One school catered for pupils aged 2–19 years with severe or profound intellectual disabilities and the other 3–11 years with moderate or severe intellectual disabilities. The two schools were federated in 2017 coming under the leadership of the same executive headteacher and both have been rated Good in their most recent Ofsted inspections. The assistant headteachers of the schools agreed to be involved after initial contact was made by the researcher and teacher participants were recruited through the assistant headteachers. The headteachers of three further special schools in the same county were invited to participate with one declining and the remaining two providing no response.

Rabiee (2004) suggested that an optimum number of focus group participants is between 6 and 10. Although the number of participants seemed large, it was felt by the assistant headteacher at one of the schools that it would be appropriate to include all teachers as participants. Similarly, the research team did not wish to exclude any eligible teachers who showed an interest in participating. All of the participants met the eligibility criteria to participate; they were qualified teachers currently working in a special school with recent experience of working with pupils on the autism spectrum with coexisting intellectual disabilities. It is important to note that in the 2019 UK Teacher Workload Survey, 73% of primary school teachers and 87% of secondary teachers surveyed stated that workload was a very serious or fairly serious problem (Walker et al., 2019). The report stated that ‘most respondents reported that they could not complete their workload within their contracted hours, that they did not have an acceptable workload, and that they did not achieve a good work-life balance’ (Walker et al., 2019). Adding to this pressure was considered unethical and, in order to ensure that teachers were not deterred from participating if they wished to take part, measures were taken to reduce time pressure and workload burden on the teachers choosing to participate. Three participants were members of the schools’ senior leadership teams and the other participants were classroom or specialist subject teachers. By using participants from the same school in each focus group, the research could be conducted on the school premises during working hours, minimising burden on teachers’ time and workload for the reasons mentioned above. For the purposes of this research, it was thought that the discussions benefited from participants being familiar with each other as they may be better able to relate to each other’s contributions and feel comfortable challenging or disagreeing (Rabiee, 2004). It was also important that the participants may feel empowered when given an opportunity to discuss assessment, as assessment is usually a prescribed process for teachers (Kitzinger, 1995).

Teachers were given an information sheet and signed a participation consent form prior to participating. Participation was entirely voluntary and teachers could withdraw at any time without giving reason.

Both focus groups were approximately 1 hour long, took place on the school premises during school time or staff meeting time and were moderated by the first author. In order to reduce additional burden and workload for the reasons described above, the length of the focus groups was limited to the staff meeting time which was provided. The moderator followed the same semi-structured focus group interview guide for both focus groups which had been refined following a pilot test. For example, in the original interview schedule a list of skills and behaviours were provided for teachers to discuss. In the pilot test, however, it became apparent that the list stifled discussion rather than encouraged it and so the decision was made to remove the list and instead allow teachers to freely discuss the areas of their choice. Teachers were asked their opinions on important progress for this specific group of pupils, barriers to learning and thoughts on assessment. The interview schedule was based on the structure set out by Vaughn et al. (1996). Although none of the questions were considered sensitive or threatening, the more simple and straightforward questions were asked first, followed by the questions that may be perceived to be more difficult. Teachers were also asked to try not to direct their answers to the moderator but, where possible, discuss their thoughts and ideas as a group. Participants were asked to let everyone who wished to say something have a chance to do so and the conversation was polite and courteous with few interruptions. The focus groups were audio recorded with notes made during the discussions in order to develop follow up question and support summaries. The moderator verbally summarised the discussion after each question and conducted participant checking at the end of the focus groups. Participants were then given the opportunity to agree with the summary, correct any misinterpretations and add information if they wished. The data were transcribed orthographically including all audible spoken words, sounds, utterances, interruptions, laughter, pauses and mumbles of agreement.

### Data analysis

The data were analysed using template analysis. Template analysis goes further than more general thematic analysis by allowing for an a priori coding template which is refined as the data analysis develops (King, 2012). Template analysis often uses four or more levels of themes and subthemes, allowing the analysis to consider the depth and detail of the data, in contrast to only one or two layers of subthemes often found in thematic analysis (Brooks et al., 2015). A six-step approach to template analysis was followed as outlined by Brooks et al. (2015).

Firstly, a priori themes were decided upon based on the previous stage of this research, the researcher’s professional knowledge and the initial engagement with the data. During the data analysis procedure, these main themes and their subthemes were revised until a final version was decided upon and the six main themes defined. The initial coding and determination of themes was conducted by the first author with discussions taking place with the second and third authors until the concept tree and final themes were agreed. As well as the verbal participant checking, the final definition of themes and the concept tree outlining subthemes were sent to a participant from each focus group for confirmation that the themes accurately reflected and covered the content of the discussions.

## Results

Six main themes were identified from the two focus group discussions with a total of four levels of themes and subthemes. The main themes were categorised as *Autism-Related Barriers and Atypical Skill Development, Overcoming Barriers, Priorities for Autistic Pupils, True Mastery, Assessing the Bigger Picture* and *Practicalities of Assessment*. The themes and subthemes are shown in Table 1 and further outlined below.

**Table 1.** Concept tree.

Theme 1. Autism-related barriers and atypical skill development 1.1 Intrinsic barriers to learning 1.1.1 Restricted and Repetitive Behaviours 1.1.1.1 Rigidity in thought 1.1.1.2 Repetitive and ritualistic behaviours 1.1.1.3 Restricted behaviours and interests 1.1.1.4 Difficulty with change and transitions 1.1.2 Physical and sensory needs 1.1.2.1 Sensory seeking 1.1.2.2 Sensory aversions and the environment 1.1.2.3 Basic physical needs 1.2 Atypical skill development resulting in barriers to learning 1.2.1 Emotional states 1.2.1.1 Heightened states including anxiety and frustration 1.2.1.2 Self-awareness and recognising emotions 1.2.1.3 Regulating emotions and behaviour 1.2.2 Learning behaviours 1.2.2.1 Attention, focus and readiness to learn 1.2.2.2 Engagement and enjoyment 1.2.2.3 Confidence and self-esteem 1.2.2.4 Risk taking and problem solving 1.2.3 Functional communication 1.2.3.1 Importance of the ability to communicate wants, needs and feelings 1.2.3.2 Difficulties due to lack of communication skills Theme 2. Overcoming barriers 2.1 Pupil-teacher relationships 2.1.1 Knowing the pupil well 2.1.2 Pupil trusting the teacher 2.2 Collaboration 2.2.1 Discussions with other teachers/teaching assistants 2.2.2 Wider collaboration between schools 2.3 Links with parents/home 2.3.1 Building relationships with parents 2.3.2 An understanding of the pupil’s home life Theme 3. Priorities for autistic pupils 3.1 Generalisation to the real world 3.1.1 Generalising skills with different people 3.1.2 Generalising skills in different settings 3.1.3 Independence and self-care skills 3.1.4 Coping in the real world 3.1.5 Limitations of teaching meaningful context	3.2 Community access/engagement 3.2.1 Being accepted in the community 3.2.2 Engaging in the wider community 3.2.3 Socially acceptable behaviours Theme 4. ‘True mastery’ 4.1 Rote learning vs real understanding 4.2 Assessment reflecting true ability 4.2.1 Missing details 4.2.2 Measuring breadth of learning 4.3 Do we know what true mastery is? 4.3.1 ‘Knowing’ a pupil can do something 4.3.2 Constantly questioning and checking mastery 4.4 Interpretation can vary 4.4.1 Precision and room for variation in judgement 4.4.2 Teachers’ differing opinions/values Theme 5. Assessing the bigger picture 5.1 Looking at pupils holistically 5.2 Personalisation/individualisation 5.2.1 Heterogeneity 5.2.2 Adapting assessment for that pupil 5.3 Importance of recording the nuances and subtleties 5.3.1 Small things can be huge achievements 5.3.2 Prioritising academic progress over learning behaviours and emotional states 5.4 Regression 5.4.1 Realities of regression 5.4.1.1 It’s negative 5.4.1.2 It’s necessary 5.4.2 Difficulties assessing regression Theme 6. Practicalities of Assessment 6.1 Nowhere to record that 6.1.1 Some progress ‘can’t’ be measured 6.1.2 Assessments don’t allow for recognition of certain progress 6.2 Autism ‘not fitting’ frameworks or systems 6.2.1 Non-linear progress and spiky profiles 6.2.2 Maintaining skills and gaps in learning 6.3 Helpful aspects of assessment 6.3.1 Next steps 6.3.2 Smaller steps

### Theme 1: ‘It’s a bit of a hindrance to his work’ – Autism-related barriers and atypical skill development

This theme incorporated the areas that teachers identified as barriers to learning for pupils on the autism spectrum along with areas which teachers felt were crucial to address before curriculum learning or more complex skills could be considered. It was divided into two subthemes; *intrinsic barriers to learning* and *atypical skill development resulting in barriers to learning*.

#### Intrinsic barriers to learning

Teachers identified intrinsic barriers to learning as those related to RRBs as well as physical and sensory needs. It is perhaps unsurprising that these two areas are linked to the diagnostic criteria for autism in DSM-V (American Psychiatric Association, 2013) and relate to specific difficulties teachers may need to address with autistic pupils.

Within this subtheme, a number of different areas linked to RRBs were considered. Rigidity in thought was mentioned and examples were given, such as a pupil not being able to use initiative in varying circumstances and only operating within particular parameters. The discussion also involved ritualistic and habitual repetitive behaviours which can interfere in learning. One participant gave the example of a pupil who needed to conduct certain rituals before they could access learning, saying, ‘*you couldn’t get anything out of her until she had done that*’. Another participant stated, ‘*it’s about managing that, saying it’s ok for you to do that but not all of the time because that is restricting your experiences in school*’. Restricted interests, transitions and difficulty with change were also mentioned by teachers from both focus groups as potentially impacting pupils’ access to learning.

Physical and sensory needs were also identified as potential barriers to learning. Both sensory seeking behaviours and sensory aversions were discussed as well as basic physical needs. Participants mentioned sensory distractions as well as the specific sensory requirements of some pupils before they could settle to learn. The diversity of sensory needs was recognised and one participant acknowledged that ‘*their sensory regulation is on a huge spectrum*’. Basic physical needs such as hunger, sleep difficulties and medical needs were also seen as possible barriers to learning, as they would for any pupil. It was identified that ‘*there seems to be some patterns…physiologically*’ for autistic pupils, with bowel difficulties given as an example. Sensory input was considered across both focus groups to be helpful for pupils who may have sensory-seeking behaviour and it was viewed as necessary for teachers to be aware of the sensory needs of individual pupils and any such difficulties pupils may be facing.

#### Atypical skill development resulting in barriers to learning

These areas were identified as skill gaps which would result in barriers to learning and included emotional regulation, learning behaviours and difficulties with functional communication. Deficits in these skill areas may result in barriers for any learner, however difficulties are likely to be exacerbated for pupils on the autism spectrum due to their specific needs.

Emotional states were discussed by many teachers throughout both focus groups as significantly impacting access to learning. Anxiety and frustration were specifically identified as problematic alongside difficulties in children recognising, regulating and coping with their emotional states. There were discussions of ‘*recognising their own emotions*’ and supporting children to ‘*cope with the situations when they go wrong*’. Anxiety and heightened emotional states may be considered consequences of other difficulties rather than barriers in themselves, however it was clear from the focus group discussions that the regulation and managing of pupils’ emotional states were one of the biggest challenges for both pupils and teachers.

Another idea that emerged from conversation involved learning behaviours. Areas discussed as making a positive difference to learning included attention skills, focus, readiness to learn, engagement, enjoyment in learning, confidence, self-esteem, risk-taking and problem solving. The discussion suggested that, although these skills are ‘expected’, they are not usually taught directly or assessed through the curriculum regardless of how necessary they are for learning to begin. One participant expressed the idea that ‘*learning isn’t just about learning subjects*’. Another teacher gave an example of the impact of small steps in these learning behaviours which actually represent great progress for the individual pupil:

We’ve had some children that won’t even come into the class and then you look, say, six months later they’re in the class…that is progress.

Communication was a final area discussed in relation to skill gaps resulting in barriers to learning. Almost all teachers across both focus groups strongly agreed that an effective form of communication, in whatever way was most suitable for that child, was necessary for learning to be able to take place. Teachers spoke of pupils being able to communicate their wants, needs and feelings and highlighted the need for the communication to be spontaneous and functional. One teacher gave the example of a pupil who used their own variations of signs to communicate which meant only staff who knew them well understood what they were trying to say. They explained, ‘*she tries to communicate and then gets really angry and then the behaviours start so there’s a barrier already with her even thinking, “oh I won’t even bother then”*’. The link between lack of functional communication abilities and other areas resulting in learning barriers, such as frustration and anxiety, was addressed throughout the discussion.

Emerging from the discussion was that the interactions between these intrinsic characteristics or needs of these pupils and their potential skill gaps provide a profile of barriers to learning that autistic children in special schools may face. The interaction of these various difficulties along with the heterogeneity of autism results in barriers which appear specific to this group of pupils.

### Theme 2: ‘Only when you are at a place where you understand that child can you really…help them to overcome their barriers’ – Overcoming barriers to learning

This theme emerged throughout both focus groups as teachers discussed factors which most helped their pupils on the autism spectrum. Three subthemes were evident; *pupil-teacher relationships*, *collaboration* and *links with parents/home*.

#### Pupil-teacher relationships

This subtheme was evident through the entire course of both discussions with group agreement on the importance of the relationship between a pupil and teacher. Teachers talked about really knowing their pupils and explained, ‘*we get to the point where we can read them well*’. Participants also talked about the relationship from the opposite perspective and highlighted the importance of the pupils knowing and trusting the teacher. The length of time it can take to build those teacher-pupil relationships was acknowledged as a difficulty, with one teacher discussing the pupils who had been in their class for 9 months and stating, ‘*I’ve only now really got to know them*’.

Further difficulties were mentioned, such as the fact that pupils may not engage with an unfamiliar staff member as they would with someone they know well. It was recognised that if there is a good relationship with teachers then the pupils are ‘*more inclined to show you what they’re capable of doing*’. There was a strong accord among teachers that knowing a pupil well could make a huge difference to their progress in accessing their learning and overcoming the barriers identified in Theme 1.

#### Collaboration

Collaboration between teachers or school staff, both within a school and between schools, was described as beneficial to support pupils and effectively assess pupil progress. Teachers commented, ‘*we do discuss it a lot as a class team*’ and ‘*sharing our expertise is key*’. Collaboration was also mentioned as a way of reducing teacher workload. Discussing pupils’ needs and progress with support staff, other teachers and between schools was considered useful as it helped creativity with teaching and behaviour management strategies as well as addressing the areas described in Theme 1.

#### Links with parents/home

Teachers across the two focus groups recognised the importance of building relationships with parents and having an understanding of pupils’ home lives. One of the focus groups highlighted this as a crucial aspect of overcoming barriers. Teachers described progress that parents were particularly pleased with, which mainly involved socially appropriate life skills such as being able to take their child on holiday or out to a restaurant. Teachers identified that more support can be provided for parents when there is an awareness of circumstances at home. There seemed to be genuine positivity from teachers when they talked about collaborating with parents, whether sharing positive news about the pupil or receiving input from parents. The benefit of consistent approaches across home and school was also mentioned when discussing pupil progress.

### Theme 3: ‘That’s a big part of what we need to teach them in school’ – Priorities for autistic pupils

Theme 3 of the discussion included other skills that teachers thought it important for their pupils on the autism spectrum to make progress with. This theme mainly incorporated the application of skills and the two subthemes included *generalisation to the real world* and *community access and engagement*. Some ideas in this theme also related to supporting parents and improving life for the pupil and family at home, showing evidence of overlap with Theme 2.

#### Generalisation to the real world

Teachers stressed the importance of their pupils being able to generalise skills. They stated that it wasn’t enough to just teach skills in school but that pupils needed to be able to ‘*transfer those skills*’ to ‘*different settings*’ and display them with ‘*a variety of adults*’. The teachers repeatedly mentioned independence and self-care when discussing priorities for their pupils and highlighted specific skills such as hanging up their coat, using the toilet appropriately and using a knife and fork. Participants talked about teaching generalisation of skills in order for pupils to being able ‘*to cope in the world*’ and preparing pupils for life after school. There were discussions about supporting pupils to be present around large groups of people and to communicate with others outside of the structured environment in which they were taught. It was also acknowledged, however, that teachers face limitations to teaching skills in meaningful contexts. Although pupils may have opportunities to access the community through school, most teaching is restricted to school settings and it can be difficult to fully prepare pupils for everything they may come across in the ‘real world’. As one teacher identified, ‘*you can’t replicate or anticipate every single situation or variation of situation that that person may come across. It’s impossible*’.

#### Community access and engagement

Within the discussion of preparing pupils to manage outside of school, teachers talked about access to and engagement in the community. Accessing the community was discussed both in terms of the pupils being able to be physically present in public areas, such as swimming pools and restaurants, and also in terms of the public accepting the presence of the children in these community spaces. A large part of this subtheme involved prioritising the teaching of socially appropriate behaviours. Teachers mentioned the difficulties that may be faced for pupils and their families if they display behaviours such as eating with their hands, not being able to sit in a public place or removing clothes. Two participants discussed an example of one such important life skill:

If they’re going to the toilet…to not come out of it with your pants around your ankles, you know, so you pull your pants up, pull your trousers up [shut the door in the first place].

The teachers talked about pupils having more opportunities in the future if they could learn skills such as these. A link was also evident with behaviours discussed in Theme 1 such as managing emotions, functional communication and RRBs.

### Theme 4: ‘We’ve assessed it this way but is that really mastery yet?’ – ‘True Mastery’

During discussion on assessment and important skills and progress, an idea seemed to filter through the conversations which we have termed ‘true mastery’. Through the discussion, teachers questioned and attempted to define ‘true mastery’ and then considered if and how it might be assessed.

#### Rote learning vs real understanding

Teachers identified that there is a difference between rote learning and real understanding. They acknowledged that, perhaps in part due to autistic pupils' restricted behaviours, skills are often learnt by rote and then performed within very specific parameters. They acknowledged that, when assessing skills, it was easy to assume that a pupil could do something with understanding when actually it was rote learnt. One teacher gave the following example:

It’s like a young child when they first learn to count…they can learn to count to five but they don’t know what five is.

The ability of teachers to distinguish between rote learning and real understanding was considered important, with one participant explaining, ‘*there’s that danger… that we teach and they learn it in a rote fashion*’. Another teacher used an example of echolalia, saying ‘*you’d think they understand what you were saying but actually they’re just repeating*’.

#### Do we know what true mastery is?

During both focus group discussions, teachers questioned how true mastery could be identified or whether it could even be defined. They questioned, for example, ‘*how do you really know when a child’s completely generalised a skill?*’ Evaluating mastery was considered an ongoing process during assessment, with some participants believing that true mastery can never be defined. Some teachers attempted to resolve this question by suggesting that there is an element of ‘just knowing’ that a child has mastered a skill. It was also suggested that the photographic and video evidence ‘proving’ a pupil has mastered a skill isn’t necessary to the extent that it is sometimes required.

#### Interpretation can vary

Participants brought up potential difficulties around variation in teachers’ interpretations. Participants acknowledged that differing opinions or values can lead to varying perceptions of the same skill and they talked about the fact that ‘*different staff members might have different thresholds*’ when assessing a skill. One participant also acknowledged that assessment can be reflective of a teacher’s abilities as well as their opinions and values. A number of participants identified, with agreement from the group, that there is pressure on teachers to show progression in assessments, regardless of circumstances, ‘*because children have always got to make progress…and you’re questioned if they don’t*’. Comments were also made about the importance of precision and uniformity in assessments, both within schools and across schools, to ensure there is a common language and understanding around the progress assessed.

#### Assessments reflecting true ability

Teachers talked around the idea that assessments don’t always reflect the true ability of a pupil. They mentioned the importance of being able to evidence breadth of learning and record the different circumstances in which a child might be able to demonstrate a skill. This subtheme linked to the subtheme of *rote learning vs true understanding*, with the suggestion that assessments often don’t allow differentiation between these types of learning. One participant explained it was sometimes difficult to determine skill level:

It might not necessarily be an indication of…whether they can do that securely every time they’re presented with that in different situations.

When talking about the different needs of pupils on the autism spectrum, another participant stated, to mumbles of agreement from the group, ‘*assessment tools don’t reflect that do they? They’re too rigid*’.

### Theme 5: ‘The actual assessments don’t tell the full story’ – Assessing the bigger picture

In both focus group discussions, teachers spoke of the ‘bigger picture’ being particularly important for autistic pupils. There was discussion around assessments needing to take account of the context and situation when demonstrating skills and allowing for individual aspects of a pupil’s learning.

#### Looking at pupils holistically

Teachers talked about pupils’ learning happening alongside other learning in the wider context of their lives. Participants highlighted that a central part of teaching at school is about ‘*developing the whole child*’. One teacher spoke of the artificiality of considering skills in isolation from this wider context. They explained:

I can pull in lots of observations that make a holistic overview of where that child is in many, many different areas because I just don’t think you learn maths like that…I think it’s a mixture of activities in a context that’s appropriate to them and the situation.

This holistic approach of looking at the whole pupil rather than looking at individual skills in isolation appeared important to teachers, particularly when it came to assessment.

#### Personalisation/individualisation

Teachers recognised the heterogeneity of autism, mentioning that autism is a spectrum condition and that each child is ‘*unique and individual*’ with a diverse range of needs. They discussed that they often need to adapt assessments to suit the needs of the pupils they teach. One example was the necessity to modify statutory speaking assessments for pupils who were pre-verbal and it was noted that the onus was on the teacher to adapt assessments accordingly. Some participants spoke of a desire for ‘*bespoke assessment systems*’ which allow an element of personalisation. One participant was sceptical of this possibility, however, and stated, ‘*a perfect catch all cover all assessment system…I can’t imagine it, not something that could cover every single child*’. Another participant responded by suggesting that it is up to the teacher to select appropriate assessments and commented, ‘*I think it’s our responsibility maybe to find other tools for certain children to make it more individual because, as you say, not one size fits all*’. One participant, however, had concerns about this in terms of teacher workload and stated reservations about teachers ‘*reinventing the wheel*’ rather than collaborating and sharing effective assessment practice within and across schools. This subtheme captured the idea that, if it could be done in a practical sense, personalisation of assessments and the option to individualise them for individual pupils would be useful.

#### Importance of recording nuances and subtleties in behaviour

Teachers were categorical in talking about how very small steps of progress could be important for a pupil and their family. When talking about skills and behaviours identified in Themes 1 and 3, seemingly small progress was described as ‘*so important*’ and ‘*really small yet massive in their achievement*’. Teachers mentioned how academic skills are often prioritised even though other progress which may seem small is often a bigger achievement and more important for that pupil. An example was given of a pupil who had managed to communicate how they felt more appropriately, even though they still had difficulties in their classroom learning. Teachers expressed how, in spite of the importance, assessments didn’t allow for the recording of such small amounts of progress.

#### Regression

The conversations around the concept of regression developed in an interesting way. In one focus group there was initially a reluctance to consider regression in skills, abilities or behaviour as something that should be recorded or assessed. Teachers asserted ‘*no, we don’t do it*’, ‘*absolutely no way we would ever be allowed to say that a child regressed*’ and ‘*it’s just so negative, isn’t it?*’ Gradually, the conversation altered after one of the participants questioned this established need to always make progress. They recognised:

If they’ve reached a certain point then the expectation is that they go to the next level. But realistically you know that they’ve actually gone backwards…that needs to be noted.

At this point, the participants began to identify that there was nowhere for teachers to show regression in current assessments and, in reality, there are ‘*always times where their learning and abilities would regress*’. Both focus groups mentioned the summer holidays as times when pupils might present with some regression in skills and behaviour. Participants also acknowledged that major changes at school or home could result in regression and they recognised the importance of identifying regression in terms of addressing the bigger picture for these pupils. One example was the extra support that was able to be provided for a family during a parent’s illness which had only become apparent to the school as a result of the child’s regression. The conversation also mentioned the difficulties of assessing regression, with teachers addressing the fact that once progress was recorded it couldn’t be ‘undone’ on many assessment systems. Some teachers said they felt confident in addressing regression through Individual Education Plans and targets. However, participants stated that few assessments that were used had a facility to show regression. The groups agreed that where regression existed, it is important to be able to show it. During this discussion, one teacher noted the necessity of distinguishing regression from lack of generalisation in skills, for example if a pupil has a change of class teacher. This links to the conversation around difficulties these pupils often have with generalising and the challenge in determining at which point skills have been mastered.

### Theme 6: ‘There’s no way of really recording or measuring that impact that we know is huge’ – Practicalities of assessment

This final theme pervaded the discussion on assessment. It incorporated some of the practicalities of assessment that were brought up by teachers including the fact that there is nowhere to record certain progress. It also encompassed the fact that spiky profiles and non-linear progress made by pupils on the autism spectrum mean they often do not fit into the usual assessment frameworks or systems. As part of this theme, teachers also identified some aspects of assessment that are helpful and useful.

#### Nowhere to record that

Throughout both discussions, participants on multiple occasions brought up the fact that there was nowhere to record some of the progress they were recognising. This was often in relation to the areas identified in the discussions on barriers to learning and teaching priorities. Teachers often expressed this in a questioning way, for example, ‘*where do I put this? Where do I write this down?*’ They mentioned that assessments often don’t cover those small aspects of progress which were identified as important to teachers, pupils and parents. One teacher spoke of the creativity needed to ‘*try to make it fit*’ in situations where progress can’t be recorded straightforwardly within an assessment system. As well as the practicality of having no way to record certain progress, teachers also suggested that there is some progress which *can’t* be measured. One participant questioned, ‘*how do you showcase how willing someone is to want to learn?*’ This was similar to the way teachers questioned whether true mastery can ever be determined.

#### Autism not fitting assessment frameworks and systems

Teachers spoke specifically about the needs of their autistic pupils and the fact that they often didn’t fit into the assessment frameworks and systems used. Teachers mentioned the ‘*spiky profile*’, the ‘*peaks and troughs*’ that are characteristic of autism, and the fact that these pupils are ‘*not linear with their progress*’. One teacher commented:

If you’re going to look at national and statutory requirements for assessment it has always been the kind of cohort with autism that have probably least best fit the P levels.

The same teacher described children on the autism spectrum with intellectual disabilities as being the ‘*worst done by*’ when it comes to assessment. Teachers also talked about the gaps in learning for these pupils and the need to revisit learning in order to maintain skills. One teacher stated that ‘*showing the pattern*’ would be better than forcing the learning profiles of autistic pupils to fit into a linear assessment framework.

#### Helpful aspects of assessment

Finally, teachers identified some aspects of assessment they found helpful and which they would like to see in future assessments. Teachers explained that it was useful when assessments signposted next steps in learning and assisted teachers to set pupil targets. Teachers spoke positively of assessments which break down progress into small steps and help them to understand the progression of a child’s skills in a particular area. One teacher mentioned identifying areas where there are interruptions to development or gaps in learning so that teachers know where further support or interventions are required. Early years teachers across both focus groups showed more satisfaction with the assessments they used and the way they assessed their pupils than the teachers who taught primary or secondary aged pupils.

## Discussion

The themes developed from the data showed that SEN teachers consider important progress for pupils on the autism spectrum to be related to barriers to learning for these pupils. RRBs, sensory needs, functional communication, recognition and regulation of emotions and learning behaviours are likely to impact upon further academic or pre-academic progress made in schools. Therefore, it is no surprise that these areas are considered priorities due to their potential impact upon education. Interestingly, the teacher participants in this study did not include or describe ‘challenging behaviour’ in itself as a barrier to learning. Instead they closely considered behaviours or skill gaps which may *result* in challenging behaviour and, in turn, affect access to learning. Teachers also identified generalisation and application of skills to the ‘real world’ as priorities. This related to the importance of supporting parents and the home life of pupils and their families. Teachers spoke about the limitations for parents when a pupil’s behaviour is not considered to be ‘socially acceptable’. The opportunity to address these areas is likely to be directly affected by the barriers to learning that were identified. Although the barriers to learning which teachers identified were discussed in the context of a school environment, it is possible, and perhaps even likely, that addressing those barriers to learning would also positively impact upon pupils’ home life and improve access to and engagement in the community for the pupils. The discussion around collaboration and relationships between teachers and parents confirmed previous research findings that these partnerships are ‘best practice’ in the education of children on the autism spectrum and impact positively upon outcomes and care (e.g. Azad and Mandell, 2016; Syriopoulou-Delli et al., 2016, p. 2).

When considering these areas in terms of assessment, teachers spoke of having skills and behaviours broken down into small steps and for assessments to recognise particularly small aspects of progress. Teachers identified that the ‘*nuances and subtleties in behaviour*’ can be of particular importance in terms of pupil progress and, therefore, are the types of progress that need to be recorded.

A number of other aspects of assessment discussed by teachers need consideration due to the potential conflict between them. Two such dichotomous areas involved the discussion around precision in assessments to minimise the variation in interpretation by different teachers contrasted with the conversation on the advantages of potential personalisation of assessments. Precise assessment items help to avoid varying interpretations by teachers when assessing pupils and this relates to the reliability of the assessment. Inter-rater reliability may therefore be important in order to ensure that different teachers or raters are assessing the same skill or behaviour. However, precision may result in the assessment being what teachers described as ‘*too rigid*’ and may require teachers to adapt the items themselves in order to fit the needs of their pupils. Personalisation may affect both the reliability of the assessment if different raters are adapting or individualising an assessment separately and the validity of the assessment if the assessment item has been personalised to a point that it no longer measures what the original item intended to measure. Linked to personalisation, teachers also spoke about the need to have a holistic overview of a pupil in order to assess their progress. They recognised that when a teacher knows a pupil well, they are more able to consider depth of learning. This supports the use of report measures in schools, where teachers assess skills based on their knowledge of the child. However, the need to know a pupil well may, again, affect reliability of an assessment. In light of the teachers’ discussion, if an element of personalisation can be added to a teacher report measure while still ensuring reliability and validity, the assessment could prove particularly helpful and useful for teachers.

Two further ideas which need to be reconciled included the need for assessments to address next steps which contrasts with the desire for an assessment to be non-linear. Linear assessments which track development, inherently show teachers what is next in the acquisition or development of a skill. Teachers acknowledged during the discussions that, due to the spiky profiles and unusual learning patterns of autistic children in special schools, learning and progress may be scattered and therefore difficult for a linear assessment tool to capture. It may be beneficial to diverge from traditional linear conceptualisations of skill development when creating assessments for these pupils and explore ways which non-linear progress can be taken into account while still providing next steps.

Finally, teachers discussed the need for assessments to acknowledge and account for regression in skills. Teachers identified that assessments often don’t allow for the recording or reporting of regression and may need to be adapted to ensure current and future progress reports are accurate. They recognised that regression may be part of the bigger picture for some pupils and can also potentially alert them to other needs that a pupil may have.

### Limitations

This research has given SEN teachers the opportunity to outline their views on barriers to learning, important progress, teaching priorities and assessment of autistic pupils in special schools. There are, however, a number of limitations of this study. Firstly, a small sample was used from two schools in one area of the United Kingdom. The discussions and themes were similar across the two schools and it is therefore encouraged that the research is replicated with SEN teachers in other areas to determine if these views are representative of those in other special schools. An additional limitation was that data on the demographics of teacher participants beyond gender and location, such as age, race and years of teaching experience, were not collected. This decision was made for a number of reasons. Firstly, minimal information was collected from participants to ensure privacy and confidentiality in line with the principle of data minimisation within the General Data Protection Regulations and this meant that data collection was limited to that which was absolutely necessary. Secondly, the time for the focus groups was limited to the length of the staff meetings and additional questionnaires would take time from the focus group discussions. Thirdly, the data and themes would not have been analysed in relation to the participant demographics as the transcripts were anonymous. Although there may have been some pertinent patterns and information in relation to the demographics of the group as a whole, including all available teachers from one school and 7 of the 10 available teachers from the second school meant that the teachers in each focus group made up a reflective sample of the teachers from these schools. A further limitation was that the focus of this research is purposely narrow; the participants were teachers from special schools and the discussions were focused upon autism. Caution should be taken if results are to be interpreted outside of this context. Finally, it may be that issues discussed were misinterpreted or lost meaning during the qualitative analysis. The possibility of this was mitigated through the use of participant checking, a reflective journal kept by the researcher and the input of the research team throughout the process.

## Conclusion and recommendations

Most SEN teachers have very good knowledge of their individual pupils’ needs and priorities for their progress. In spite of this, teachers are not often given a voice when it comes to the way that progress is identified and recorded. In order to ascertain the face and content validity of assessment tools while also ensuring that assessments are useful and valued by teachers, it is imperative that teachers have input during the assessment development stages and that any concerns and preferences about the assessment content and method are taken into account. It is recommended that teachers are involved in all aspects of the assessment development process, particularly prior to the development of an assessment when defining parameters and considering the purposes for which the assessment will be used. Teachers should have input into the items to be used within assessments, and their judgements about the utility of an assessment should be sought and considered. It may be useful for further research in this area to consider the link between assessment and effective interventions in these priority teaching areas for pupils on the autism spectrum who attend special schools. It is also recommended that new teacher assessments take account of the ideas discussed above, notably potential elements of personalisation, accounting for regression and the non-linear progress often shown by autistic pupils as well as the possibility of varying teacher interpretations when assessing pupils. Implications of this research include the opportunity for those developing teacher and school assessments to consider and include the priorities of the specialists who work most closely with pupils on the autism spectrum. Assessment requirements specific for the needs for these pupils were highlighted by the teachers and these can be used to determine effective and useful assessment practice in special schools. Unhelpful and ineffective assessments can measure irrelevant skills, add to teacher workload and waste valuable teaching and learning time. It is necessary, therefore, for assessments to be developed for the unique needs of pupils on the autism spectrum, for the specific context of use in special schools and with input from the individuals who use them – the teachers.

## Supplemental Material

Supplemental Material, Appendix_1._Semi_Structured_Interview_Schedule - ‘There isn’t a checklist in the world that’s got that on it’: Special needs teachers’ opinions on the assessment and teaching priorities of pupils on the autism spectrum Click here for additional data file. Supplemental Material, Appendix_1._Semi_Structured_Interview_Schedule for ‘There isn’t a checklist in the world that’s got that on it’: Special needs teachers’ opinions on the assessment and teaching priorities of pupils on the autism spectrum by Melanie Howell, Jill Bradshaw and Peter E Langdon in Journal of Intellectual Disabilities
