# Supplementary material for: ‘There isn’t a checklist in the world that’s got that on it’: Special needs teachers’ opinions on the assessment and teaching priorities of pupils on the autism spectrum
Source: J Intellect Disabil. 2020 Dec 16;26(1):211–26. doi: 10.1177/1744629520972901 (PMC9016664; doi:10.1177/1744629520972901)
Supplement: Supplemental Material, Appendix_1._Semi_Structured_Interview_Schedule - ‘There isn’t a checklist in the world that’s got that on it’: Special needs teachers’ opinions on the assessment and teaching priorities of pupils on the autism spectrum [file Appendix_1._Semi_Structured_Interview_Schedule.docx]

Semi-Structured Focus Group Interview Schedule

1. Introduction
2. Welcome
3. Background
4. Purpose of research
5. Guidelines
6. Questions
7. Questions
8. Initial question should be general and less threatening
9. Establish More Difficult Questions
10. Wrap Up
11. Identify and organise the major themes from responses
12. Ensure that any conversational points not completed are mentioned
13. Closing Statements
14. Request anonymity of information
15. Answer any remaining questions
16. Express thanks

Establish Easy and Non-Threatening Questions

1. What skills or behaviours do you think it is most important to see your pupils with autism making progress with? Please consider broad areas as well as specific skills. *(10 minutes)*
2. What are the barriers to accessing learning for your pupils with autism? *(10 minutes)*
3. Which of these skills and behaviours that we have been discussing do you find it difficult to show progress in using current assessments? *(5 minutes)*

Establish More Difficult Questions

1. What are the features of a good assessment tool for you as a teacher? Please try to ignore preconceptions you have about what is necessary or assessments you currently use and think about what would be ideal for you. *(10 minutes)*
2. Of all the ideas we have discussed today, what do you think is the most important? *(10 minutes)*
3. Wrap Up

Identify and organise the major themes from responses: *(5 minutes)*

1. Is there anything final anyone would like to add to my summary or has anything important been missed? *(5 minutes)*

Closing Statements
